# NCX1 coupled with TRPC1 to promote gastric cancer via Ca^2+^/AKT/β-catenin pathway

**DOI:** 10.1038/s41388-022-02412-9

**Published:** 2022-07-26

**Authors:** Hanxing Wan, Nannan Gao, Wei Lu, Cheng Lu, Jun Chen, Yimin Wang, Hui Dong

**Affiliations:** 1 Department of Pharmacology, School of Pharmacy, Qingdao University Medical College, #1 Ningde Road, Qingdao, 266073 China; 2 Department of Gastroenterology, Xinqiao Hospital, Army Medical University, Chongqing, 400037 China; 3 Department of General Surgery, First Hospital of Qinhuangdao, Qinhuangdao, Hebei China; 4 Department of Medicine, University of California, San Diego, CA USA

**Keywords:** Colorectal cancer, Ion channel signalling

## Abstract

Plasma membrane Na^+^/Ca^2+^ exchanger 1 (NCX1) is a bidirectional ion transporter to operate in Ca^2+^ entry or exit modes, and TRPC1 is Ca^2+^-permeable channel. Both NCX1 and TRPC1 play critical roles in maintaining cytosolic free Ca^2+^ ([Ca^2+^]_cyt_) homeostasis in mammalian cells. Although either TRPC1 channel or Ca^2+^ entry mode of NCX1 is implicated in some tumorigenesis, it has not been explored if a coordination of NCX1 and TRPC1 involves in the pathogenesis of *H. pylori-*associated human gastric cancer (GC). Here we found the protein expression of NCX1 was significantly enhanced in human GC specimens, which correlated with tumor progression and poor survival in GC patients. TRPC1 and NCX1 were parallelly enhanced, co-localized and bound in human GC cells. By a functional coupling, TRPC1 drives NCX1 to the Ca^2+^ entry mode, raising [Ca^2+^]_cyt_ in GC cells. Moreover, CaCl_2_, *H. pylori* and their virulence factors all enhanced expressions and activities of NCX1 and TRPC1, and evoked aberrant Ca^2+^ entry to promote proliferation, migration, and invasion of GC cells through AKT/β-catenin pathway. Tumor growth and metastasis also depended on the enhanced expression of NCX1 in subcutaneously xenografted GC mouse model. Overall, our findings indicate that TRPC1/NCX1 coupling may promote *H. pylori-*associated GC through the Ca^2+^/AKT/β-catenin pathway. Since the Ca^2+^ exit mode and the Ca^2+^ entry mode of NCX1 play different roles under mostly physiological and pathological conditions respectively, targeting TRPC1/NCX1 coupling could be a novel strategy for selectively blocking Ca^2+^ entry mode to potentially treat digestive cancer with less side effect.

## Introduction

Since gastric cancer (GC), one of the leading causes of cancer-related death worldwide, is difficult to cure once it metastasizes [1], it is urgent to explore early diagnostic markers and novel therapeutic targets responsible for GC. Helicobacter pylori (*Hp*) infection in the stomach is a well-known risk factor for GC and ammonia/ammonium is the major *Hp* virulence factor [2], but their pathogenesis in GC is still obscure. Therefore, it is critical to elucidate molecular pathogenesis of *Hp-*associated GC. Cytosolic free Ca^2+^ ([Ca^2+^]_cyt_) is a pivotal second messenger in eukaryotic cells to maintain critical cellular processes, including the energetic metabolism, cell signaling, and cell motility, etc [3–5]. Numerous findings indicate that aberrant [Ca^2+^]_cyt_ signaling is involved in GC, though the occurrence and progression of cancer are complex [6–8]. Since membrane Ca^2+^-permeable channels and transporters play important roles in the regulation of [Ca^2+^]_cyt_, their aberrant expression and function are associated with GC development [9–11].

The Na^+^/Ca^2+^ exchanger (NCX) is a bidirectional transporter that induces Ca^2+^ efflux (when operating in Ca^2+^ exit mode), or Ca^2+^ influx (when operating in Ca^2+^ entry mode), depending on the electrochemical gradient of the substrate ions and membrane potentials [12]. Three different protein isoforms of NCX were described [12], NCX1 has a broad expression in multiple organs, including the heart, kidney, and gastrointestinal (GI) tract, *etc*, whereas NCX2 is mainly found in the brain but NCX3 mostly in brain and skeletal muscle [13, 14]. NCX has been investigated predominately in human brain, heart and kidney, and the therapeutic potentials of its modulators are also emerging for the related disease. However, the molecular and functional aspects of NCX in GI organs, especially in GI cancer are scarce although it is involved in aberrant [Ca^2+^]_cyt_ homeostasis in other cancer cells [15]. It has been shown that NCX1 is expressed in the rat small intestine [16]. Although the expression and function of NCX were reported in human gastric smooth muscle cells and myofibroblasts [17], they have not been explored in gastric epithelium. Furthermore, emerging evidence suggests a pathogenesis role of NCX in glioblastoma, melanoma, and ovary carcinoma [15]. We and others also revealed a role of NCX1 in esophageal squamous cell carcinoma and hepatocellular carcinoma [18, 19], but its role in the adenocarcinoma of GI tract has not been explored so far.

Transient receptor potential canonical (TRPC) channels as Ca^2+^-permeable channels are ubiquitously expressed in various cell types, including GI epithelial cells to regulate [Ca^2+^]_cyt_ homeostasis [20]. Among seven members of TRPC subfamilies, TRPC1 is crucial for metastasis by epithelial-mesenchymal transition (EMT) activation in several kinds of tumors [21, 22]. We reported previously that TGF-β−induced Ca^2+^ entry via TRPC1/NCX1 coupling to modulate Ca^2+^-mediated motility of human pancreatic duct cells [23]. Although TRPC1 is highly expressed in human GC to likely promote GC progression [24], it is currently unknown whether TRPC1 alone or its coupling with NCX1 contributes to this process. Therefore, in the present study, we sought to investigate if NCX1 and TRPC1 are simultaneously involved in GC; and if so, what the underlying molecular mechanisms are.

## Results

### Enhanced NCX1 expression in human primary GC tissues

**Fig. 1:**
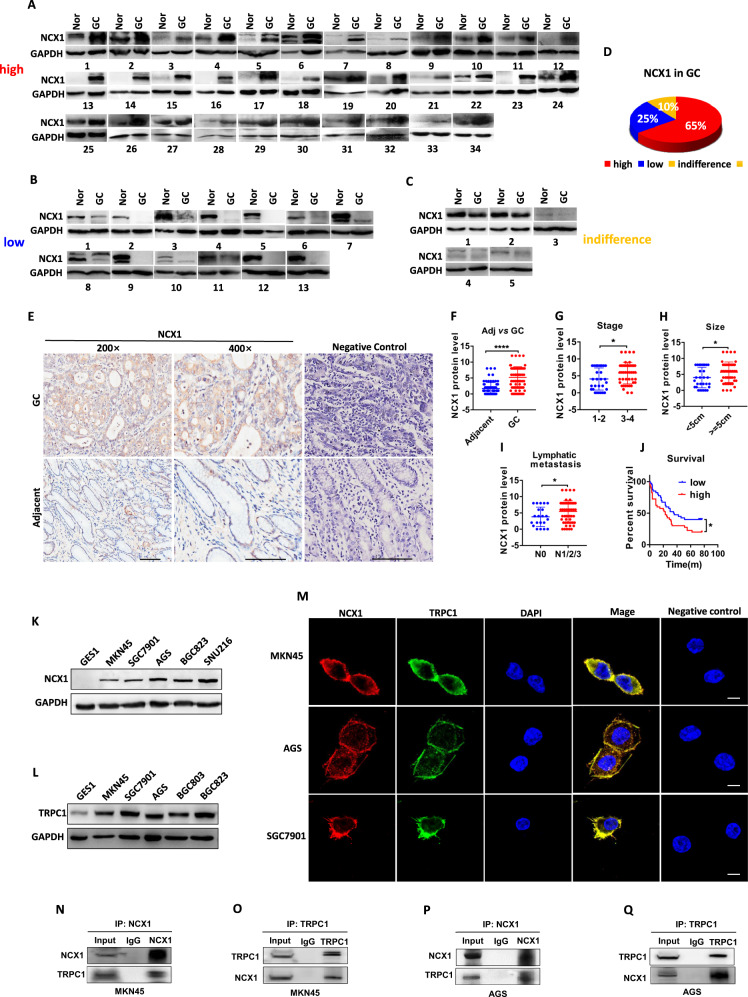
The enhanced expression of NCX1 and TRPC1 in human primary gastric cancer tissues and cells. Western blot analysis applied to compare the expression levels of NCX1 proteins between gastric cancer (GC) tissues and adjacent normal (Nor) tissues from 52 GC patients: 34 pairs with high expression (**A**), 13 pairs with low expression (**B**), and 5 pairs without difference (**C**). **D** Summary data showing the percentage of high, low and indifference of NCX1 expression in GC tissues compared to adjacent tissues. **E**, **F** Representative and summary data of immunohistological staining on NCX1 proteins in GC tissues compared to adjacent tissues. Scale bar=100 μm for each image. Negative control: without primary antibody. (^****^P < 0.0001, *n* = 80 patients). Relative NCX1 protein levels in GC tissues from the patients with different stages (**G**), tumor sizes (**H**), and lymphatic metastasis (**I**). (^*^P < 0.05, *n* = 80 patients). **J** Kaplan–Meier analysis of survival ratio of GC patients with low and high NCX1 expression levels (^*^P < 0.05, *n* = 80 patients). **K**, **L** Western blot analysis of NCX1 and TRPC1 protein levels in GES1 and GC cell lines. **M** Immunofluoresence staining images of NCX1 and TRPC1 proteins with primary antibody and without the antibody (negative control) in MKN45, AGS and SGC7901 cells. Scale bar=10 μm for each image. **N–Q** Co-immunoprecipitation showing the binding of NCX1 and TRPC1 proteins in GC cells.

Due to the lack of information on NCX1 expression in the stomach of normal subjects and GC patients, we first collected human primary GC tissues and corresponding adjacent tissues to compare NCX1 expression. By applying western blotting analysis, total 52 pairs of fresh gastric tissues obtained from GC patients were compared. As shown in Fig. 1, 34 pairs had higher NCX1 protein expression in human GC tissues than in adjacent tissues (Fig. 1A), accounting for 65% of the total (Fig. 1D). In contrast, 13 pairs had lower NCX1 expression in GC tissues (Fig. 1B), accounting for 25% (Fig. 1D). However, 5 pairs had no difference (Fig. 1C), accounting for 10% (Fig. 1D). Therefore, NCX1 protein expression was enhanced in human primary GC tissues.

Second, immunohistochemstry study was applied to human gastric tissues from 80 GC patients. Among these patients, their average age was 64 years old, 76% was male, 60% was diagnosed with advanced-stage (III/IV), and 74% had lymphatic metastasis (Supplementary Table 1). As shown in Fig. 1E, F, the protein expression of NCX1 was markedly enhanced in GC tissues compared to their adjacent tissues, but staining was not detected in the negative control, indicating a specific staining to NCX1 proteins. Third, the association between NCX1 expression and clinicopathologic parameters of GC progression was subsequently analyzed. As shown in Fig. 1G–I, the up-regulation of NCX1 expression was correlated with advanced clinical stage, large tumor size, and lymphatic metastasis. Furthermore, Kaplan-Meier analysis showed that the GC patients with high NCX1 expression had a poor prognosis, but those with low expression had a better prognosis (Fig. 1J). Altogether, the close association between NCX1 expression and clinicopathologic parameters strongly suggests an oncogenic role for NCX1 in human GC.

### Co-localization and binding of the enhanced NCX1 and TRPC1 in human GC cells

Since enhanced expression of TRPC1 was closely related to worse prognosis and exacerbated EMT in GC [24, 25], we first compared the expression of either TRPC1 or NCX1 proteins between 5 human GC cell lines and 1 normal gastric epithelial cell line (GES1). As shown in Fig. 1K, the expression level of NCX1 proteins was markedly enhanced in all GC cells compared to GES1 cells. Similarly, the expression level of TRPC1 proteins was also markedly enhanced in all GC cells compared to GES1 cells (Fig. 1L), suggesting both NCX1 and TRPC1 are expressed parallelly in GC cells and normal cells. Second, we performed immunofluorescence analysis to further study the expression and localization of NCX1 and TRPC1 proteins in human GC cells. As shown in Fig. 1M, both NCX1 and TRPC1 proteins were confirmed to express parallelly in 3 GC cell lines, but non-specific staining was undetected in the negative control without primary antibody. Moreover, both NCX1 and TRPC1 proteins were predominately expressed and co-localized on the plasma membrane of GC cells (Fig. 1M). Finally, our coimmunoprecipitation study clearly showed the binding of NCX1 and TRPC1 in 2 GC cell lines (Fig. 1N–Q). Therefore, the expression of NCX1 and TRPC1 is not only up-regulated but also co-localized and bound on the plasma membrane of human GC cells.

### NCX1 activation promotes proliferation, migration and invasion of human GC cells in vitro

**Fig. 2:**
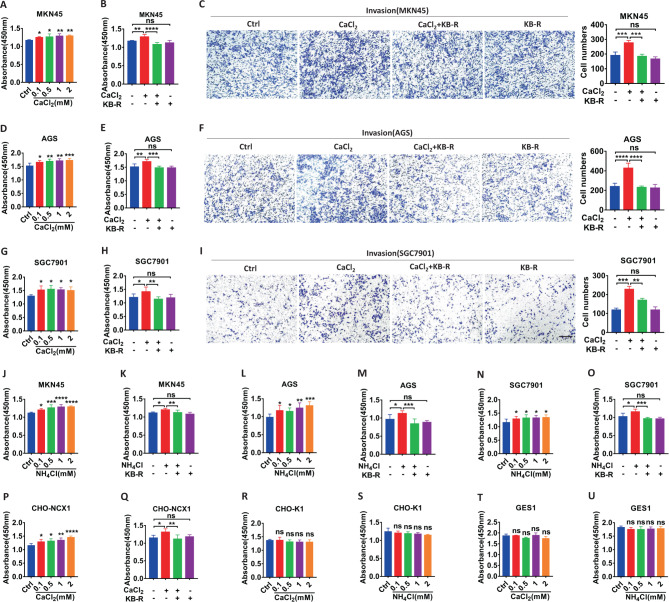
NCX1 activation promotes proliferation and invasion of human GC cells. Dose-dependently enhanced proliferation of CaCl_2_ (0.1-2 mM) in MKN45 (**A**), AGS (**D**), and SGC7901 (**G**) cells. The inhibitory effect of KB-R7943 (KB-R, 1 μM in MKN45, 4 μM in AGS, 8 μM in SGC7901) on CaCl_2_ (1 mM)-induced proliferation (**B**, **E**, **H**) and invasion (**C**, **F**, **I**) of GC cells, Scale bar=200 μm for each image. Dose-dependently enhanced proliferation of NH_4_Cl (0.1-2 mM) in MKN45 (**J**), AGS (**L**), and SGC7901 (**N**) cells, and the inhibitory effect of KB-R7943 on NH_4_Cl (1 mM)-induced proliferation of MKN45 (**K**), AGS (**M**), and SGC7901 (**O**) cells. **P**, **Q** Dose-dependently enhanced proliferation of CaCl_2_ (0.1-2 mM) in CHO-NCX1 with NCX1 overexpression, and the inhibitory effect of KB-R7943 (0.2 μM) on CaCl_2_ (1 mM)-induced proliferation of CHO-NCX1 cells. **R–U** No effects of CaCl_2_ (0.1–2 mM) and NH_4_Cl (0.1–2 mM) on proliferation of CHO-K1 without NCX1 overexpression and GES1 cells. (^*^P < 0.05, ^**^P < 0.01, ^***^P < 0.001, ^****^P < 0.0001, *n* = 3; ns, no significant differences).

To examine the role of NCX1 in GC, we first determined the cell proliferation of 3 human GC cell lines commonly used in the literature (MKN45, AGS and SGC7901). The varying concentrations of CaCl_2_ were applied to stimulate the Ca^2+^ entry mode of NCX1 since no selective activators of NCX1 are commercially available so far [26]. CaCl_2_ at the concentrations of 0.1–2 mM, dose-dependently promoted proliferation of all GC cells (Fig. 2A, D, G), which was attenuated by KB-R7943 (Fig. 2B, E, H) and SN-6 (Supplementary Fig. 1A, B), the selective inhibitors for the Ca^2+^ entry mode of NCX1. The concentrations of KB-R7943 were chosen in the light of the different sensitivity of GC cell proliferation to the drug (Supplementary Fig. 2A–D). Similarly, CaCl_2_ dose-dependently promoted proliferation of CHO cells with NCX1 overexpression (CHO-NCX1) (Fig. 2P), which was attenuated by KB-R7943 (Fig. 2Q). In contrast, CaCl_2_ could not influence proliferation of CHO cells (CHO-K1) (Fig. 2R) and GES1 cells without NCX1 expression (Fig. 2T). Therefore, CaCl_2_ promotes GC cell proliferation most likely via activating the Ca^2+^ entry mode of NCX1.

**Fig. 3:**
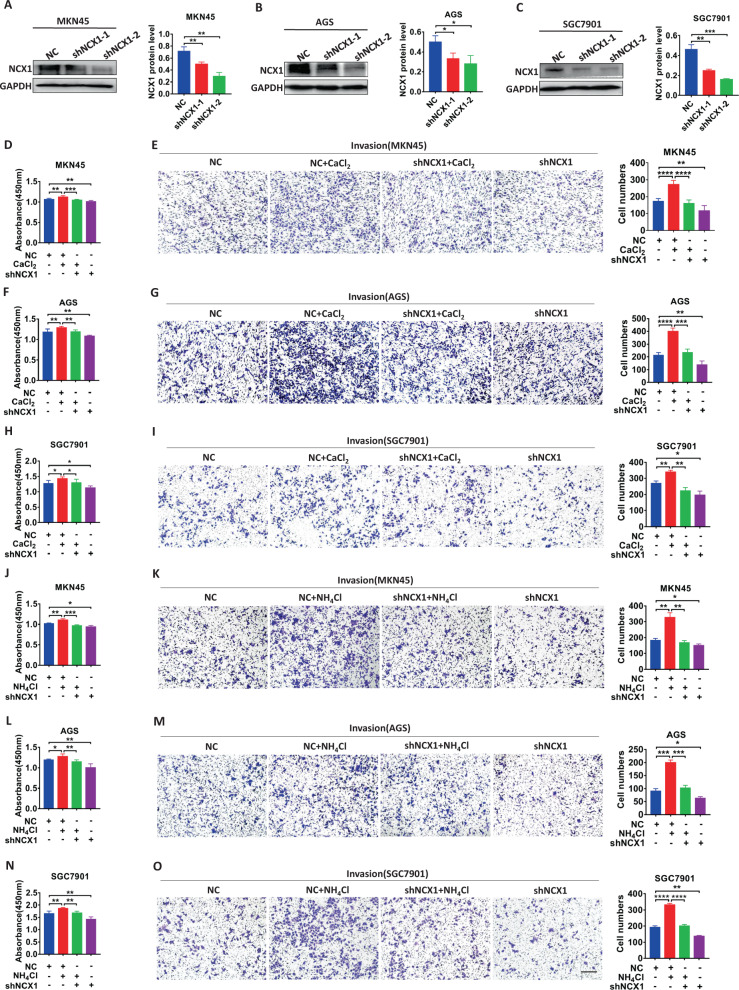
CaCl_2_ and NH_4_Cl promote proliferation and invasion of human GC cells through NCX1 activation. Representative images of NCX1 protein expression in GC cells with NCX1 knockdown and summary data of NCX1 protein levels in MKN45 (**A**), AGS (**B**), and SGC7901 (**C**) cells (^*^P < 0.05, ^**^P < 0.01, ^***^P < 0.001, *vs*. NC, *n* = 3). The effect of shNCX1 on CaCl_2_ (1 mM)-induced proliferation (**D**, **F**, **H**) and invasion (**E**, **G**, **I**) of GC cells. The effect of shNCX1 on NH_4_Cl (1 mM)-induced proliferation (**J**, **L**, **N**) and invasion (**K**, **M**, **O**) of GC cells. Scale bar = 200 μm for each image. (^*^P < 0.05, ^**^P < 0.01, ^***^P < 0.001, ^****^P < 0.0001, *n* = 3; ns, no significant differences).

Although NCX1 enhanced migration and invasion of hepatocellular carcinoma [19], its contribution to GC progression is unknown. Second, we examined the role of NCX1 in migration and invasion of human GC cells. Cell scratch test showed that CaCl_2_ promoted migration of MKN45 and AGS cells, which was attenuated by KB-R7943 (Supplementary Fig. 3A, B). Moreover, transwell assays showed that CaCl_2_ promoted migration (Supplementary Fig. 3C, D) and invasion (Fig. 2C, F, I) of MKN45, AGS and SGC7901 cells, which were attenuated by KB-R7943 (Supplementary Fig. 3C, D and Fig. 2C, F, I). Finally, after shNCX1 was applied to successfully knock down the protein expression of NCX1 in GC cells (Fig. 3A–C), CaCl_2_-induced cell proliferation (Fig. 3D, F, H), migration (Supplementary Fig. 4A–C) and invasion (Fig. 3E, G, I) were all inhibited. Taken together, NCX1 plays a critical role in GC cell proliferation, migration and invasion.

### *Hp* virulence factor promotes GC cell proliferation, migration and invasion via NCX1 activation

Since *H. pylori* infection is a pivotal risk factor for tumorigenesis of GC and ammonia/ammonium is a major *H. pylori* virulence factor, NH_4_Cl was applied to the present study as a well-known ammonia/ammonium [2]. As shown in Fig. 2, like CaCl_2_, NH_4_Cl dose-dependently (0.1–2 mM) promoted proliferation of MKN45, AGS and SGC7901 cells (Fig. 2J, L, N), which was attenuated by KB-R7943 (Fig. 2K, M, O). In contrast, NH_4_Cl did not affect proliferation of CHO-K1 (Fig. 2S) and GES1 cells without NCX1 expression (Fig. 2U). Similarly, NH_4_Cl-induced cell proliferation (Fig. 3J, L, N), migration (Supplementary Fig. 4D–F) and invasion (Fig. 3K, M, O) were all inhibited by shNCX1. Therefore, *Hp* virulence factor promotes GC cell proliferation, migration and invasion via the Ca^2+^ entry mode of NCX1.

### CaCl_2_, *Hp* and their virulence factors enhance NCX1 expression in GC cells

**Fig. 4:**
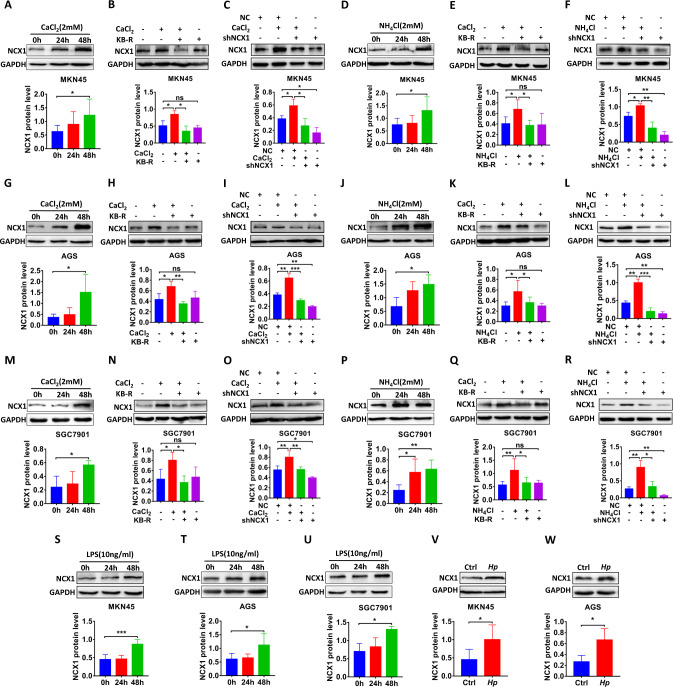
CaCl_2_, *Hp* and virulence factors enhance NCX1 expression in human GC cells. **A**, **G**, **M** Representative time courses of CaCl_2_ (2 mM)-enhanced NCX1 protein expression in GC cells. Inhibitory effect of KB-R7943 (KB-R, 1 μM in MKN45, 4 μM in AGS, 8 μM in SGC7901) (**B**, **H**, **N**) or shNCX1 (**C**, **I**, **O**) on CaCl_2_ (2 mM)-enhanced NCX1 expression in GC cells. **D**, **J**, **P** Representative time courses of NH_4_Cl (2 mM)-enhanced NCX1 protein expression in GC cells. Inhibitory effect of KB-R7943 (**E**, **K**, **Q**) or shNCX1 (**F**, **L**, **R**) on NH_4_Cl (2 mM)-enhanced NCX1 protein expression in GC cells. **S**, **T**, **U** Representative time courses LPS (10 ng/ml)-enhanced NCX1 protein expression in GC cells. **V**, **W** Representative time courses *H. pylori*-enhanced NCX1 protein in GC cells. (^*^P < 0.05, ^**^P < 0.01, ^***^P < 0.001, *n* = 3; ns, no significant differences).

After demonstrating the promotion of CaCl_2_ and *Hp* virulence factors on cell proliferation, migration and invasion, we examined if they also affect NCX1 expression in GC cells. Indeed, CaCl_2_ enhanced NCX1 expression in MKN45, AGS and SGC7901 cells (Fig. 4A, G, M), which was attenuated by either KB-R7943 (Fig. 4B, H, N) or shNCX1 (Fig. 4C, I, O). Similarly, *H. pylori* virulence factor NH_4_Cl- enhanced NCX1 expression in GC cells (Fig. 4D, J, P) was attenuated by either KB-R7943 (Fig. 4E, K, Q) or shNCX1 (Fig. 4F, L, R). Moreover, another *H. pylori* virulence factor lipopolysaccharide (LPS) [27, 28] enhanced NCX1 expression in GC cells (Fig. 4S, T, U). Finally, *H. pylori* per se also enhanced NCX1 expression (Fig. 4V, W) after co-culturing with GC cells for 24 h. However, CaCl_2_, NH_4_Cl, LPS and *H. pylori* all did not affect NCX1 expression in GES1 cells as a negative control (Supplementary Fig. 5A–D). Taken together, these data strongly suggest that like CaCl_2_, *H. pylori* per se and their virulence factors promote GC through enhancing NCX1 expression as well.

### NCX1 coordinates with TRPC1 to promote GC cell proliferation and migration

**Fig. 5:**
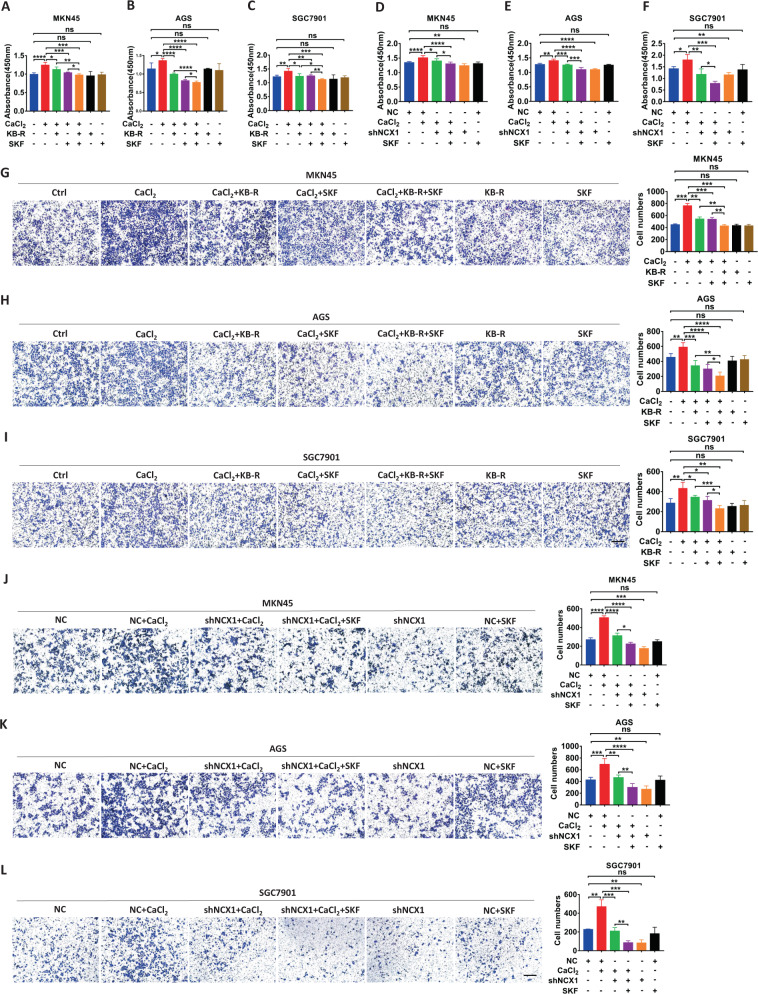
NCX1 coordinates with TRPC1 to promote proliferation and migration of human GC cells. Summary data showing the inhibitory effects of either KB-R7943 (KB-R, 1 μM in MKN45, 4 μM in AGS, 8 μM in SGC7901), SKF96365 (SKF, 1 μM) or KB-R plus SKF on CaCl_2_ (1 mM)-enhanced proliferation (**A**–**C**) and migration (**G**–**I**) of GC cells. **D–F**, **J–L** Summary data showing the inhibitory effect of either shNCX1, SKF96365 (SKF, 1 μM) or shNCX1 plus SKF on CaCl_2_ (1 mM)-enhanced proliferation and migration of GC cells. Scale bar = 200 μm for each image. (^*^P < 0.05, ^**^P < 0.01, ^***^P < 0.001, ^****^P < 0.0001, *n* = 3; ns, no significant differences).

TRPC family is a potential partner for the Ca^2+^ entry mode of NCX1 [29]. Among them, TRPC1 and TRPC6 were highly expressed in human GC to play an oncogenic role in GC progression [24, 30]. We therefore focused on a possible coupling of TRPC1/6 and NCX1 in GC development. TRPC6 antagonist SAR7334 did not affect CaCl_2_-promoted GC cell proliferation (Supplementary Fig. 1C, D), excluding the involvement of TRPC6. However, as shown in Fig. 5, CaCl_2_-induced GC cell proliferation and migration were attenuated by either KB-R7943 or a TRPC1 blocker SKF96365, which concentrations were chosen based on its sensitivity (Supplementary Fig. 2E–G). Moreover, the CaCl_2_-induced cell proliferation and migration were further attenuated by a combination of the selective inhibitors for both NCX1 and TRPC1 (Fig. 5A–C, G–I). Similarly, the CaCl_2_-induced cell proliferation and migration were further attenuated by a combination of shNCX1 plus SKF96365 (Fig. 5D–F, J–L). Taken together, these data strongly suggest that NCX1 coordinate with TRPC1 to promote GC cell proliferation and migration.

### *Hp* virulence factor could stimulate TRPC1 channels to trigger Ca^2+^ entry mode of NCX1 in GC cells

**Fig. 6:**
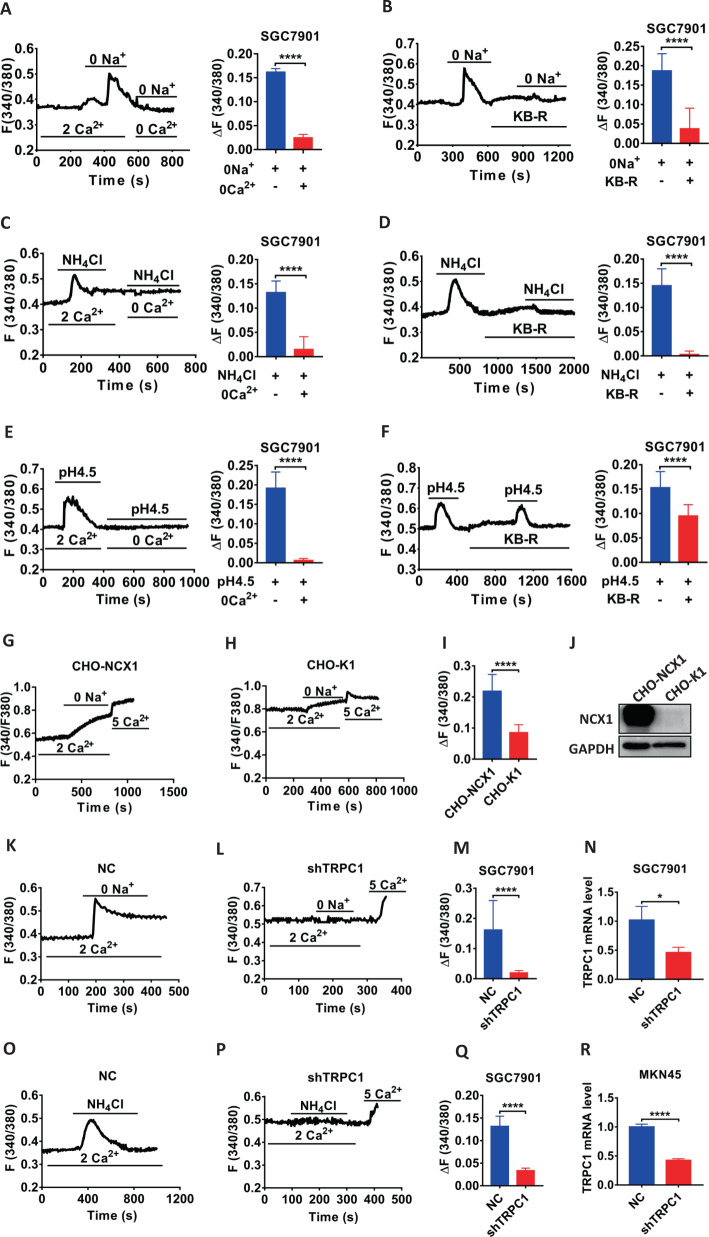
*Hp* virulence factor and acid stimulate NCX1/TRPC1 coupling in human GC cells. Summary tracings of [Ca^2+^]_cyt_ time courses in response to extracellular 0 Na^+^ (**A**), NH_4_Cl (5 mM) (**C**) and pH 4.5 (**E**) in the presence of extracellular 2 Ca^2+^ or 0 Ca^2+^ (left). Summary data showing the peaks of 0 Na^+^, NH_4_Cl and pH 4.5-increased [Ca^2+^]_cyt_ signaling in SGC7901 cells (right) (**A**, *n* = 20 cells; **C**, *n* = 11 cells; **E**, *n* = 11 cells, ^****^P < 0.0001). Summary tracings of [Ca^2+^]_cyt_ time courses in response to 0 Na^+^ (**B**), NH_4_Cl (5 mM) (**D**) and pH 4.5 (**F)** in the presence of 2 Ca^2+^ or 2 Ca^2+^ plus KB-R7943 (KB-R, 30 μM) (left). Summary data showing the peaks of 0 Na^+^, NH_4_Cl and pH 4.5-increased [Ca^2+^]_cyt_ signaling in SGC7901 cells (right) (**B**, *n* = 20 cells; **D**, *n* = 23 cells; **F**, *n* = 26 cells, ^****^P < 0.0001). Summary tracings of [Ca^2+^]_cyt_ time courses in response to 0 Na^+^ in the presence of 2 Ca^2+^ or 5Ca^2+^ in CHO-NCX1 (**G**) and CHO-K1 (**H**) cells. **I** Summary data showing the peaks of 0 Na^+^-increased [Ca^2+^]_cyt_ signaling described as in **G**, **H** (**G**, *n* = 23 cells; **H**, *n* = 17 cells, ^****^P < 0.0001). **J** Western blot analysis of NCX1 protein levels in CHO-NCX1 and CHO-K1 cells. Summary tracings of [Ca^2+^]_cyt_ time courses in response to 0 Na^+^ (**K**, **L**) and NH_4_Cl (5 mM) (**O**, **P**) in the presence of 2 Ca^2+^ in NC (**K**, **O**) or shTRPC1 (**L**, **P**) of SGC7901 cells. Summary data showing the peaks of 0 Na^+^ (**M**) or NH_4_Cl (**Q**)-increased [Ca^2+^]_cyt_ signaling described as in **K**, **L**, **O**, **P** (*n* = 11 cells, ^****^P < 0.0001). **N**, **R** RT-PCR analysis of TRPC1 mRNA levels in NC and shTRPC1 of GC cells (*n* = 3, ^*^P < 0.05, ^****^P < 0.0001). Each one is 3 independent experiments with similar results.

We next applied cell Ca^2+^ imaging to determine if NCX1 operates in Ca^2+^ entry mode to induce [Ca^2+^]_cyt_ increase in GC cells. First, extracellular 0 Na^+^ that triggers Ca^2+^ entry mode of NCX1 significantly induced [Ca^2+^]_cyt_ signaling in Ca^2+^-containing solutions but not in Ca^2+^-free solutions (Fig. 6A). Second, 0 Na^+^-induced [Ca^2+^]_cyt_ signaling in Ca^2+^-containing solutions was abolished by KB-R7943 (Fig. 6B). Third, 0 Na^+^ also markedly increased [Ca^2+^]_cyt_ signaling in CHO-NCX1 cells with NCX1 overexpression (Fig. 6G, I, J), but not in CHO-K1 cells without NCX1 overexpression (Fig. 6H–J). These data strongly support NCX1 operates in Ca^2+^ entry mode in GC cells like in CHO-NCX1 cells. We examined if NH_4_Cl and the local acidic micro-environment in *Hp* infection-induced chronic inflammation and tumorigenesis could stimulate NCX1 activity. Like 0 Na^+^, NH_4_Cl and acid (pH 4.5) indeed had similar stimulation on Ca^2+^ entry mode of NCX1 in SGC7901 cells (Fig. 6C–F).

We further examined if TRPC1/NCX1 coupling mediates Ca^2+^ signaling in GC cells since NCX1 operation in Ca^2+^ entry mode requires Na^+^ entry via TRPC channels [29]. After shTRPC1 successfully knocked down TRPC1 expression in GC cells (Fig. 6N, R), both 0 Na^+^- and NH_4_Cl-induced Ca^2+^ signaling was almost abolished (Fig. 6K–M, O–Q). These data verify that *Hp* virulence factor induces Ca^2+^ signaling via TRPC1/NCX1 coupling in GC cells.

### NCX1 activation promotes GC through AKT/β-catenin pathway

**Fig. 7:**
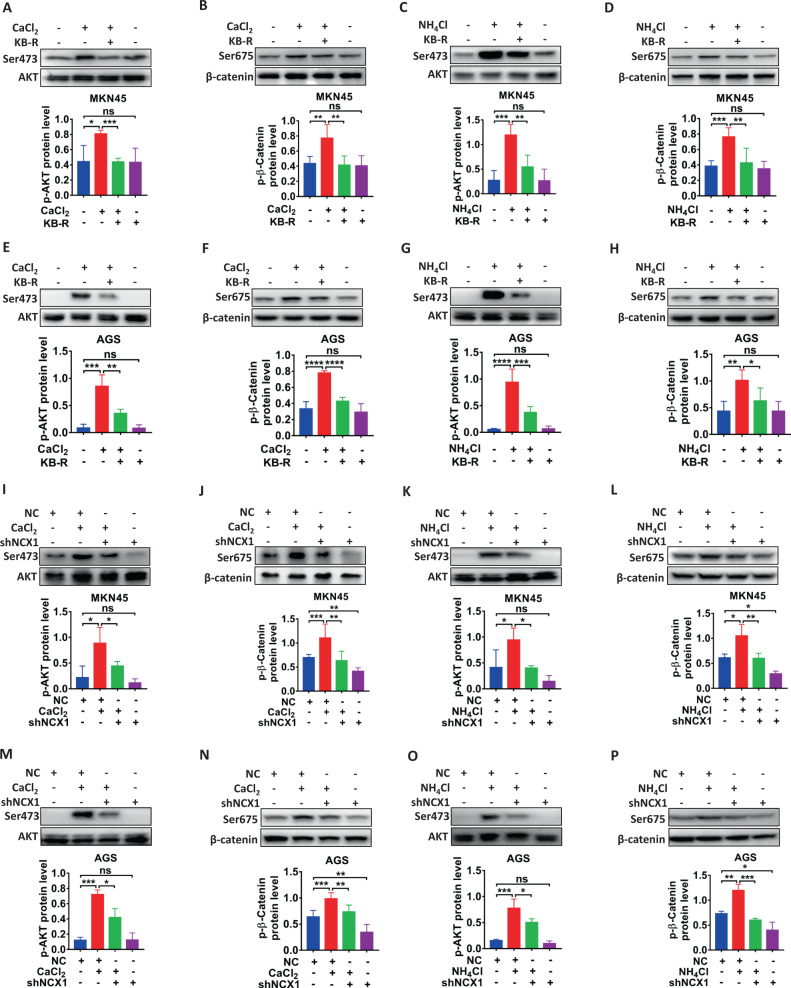
NCX1 activation induces phosphorylation of AKT and β-catenin in human GC cells. **A**, **B**, **E**, **F** Inhibitory effect of KB-R7943 (KB-R, 1 μM in MKN45, 4 μM in AGS) on CaCl_2_ (2 mM)-induced AKT and β-catenin phosphorylation in MKN45 and AGS cells. **C**, **D**, **G**, **H** Inhibitory effect of KB-R7943 on NH_4_Cl (2 mM)-induced AKT and β-catenin phosphorylation in MKN45 and AGS cells. **I**, **J**, **M**, **N** Inhibitory effect of shNCX1 on CaCl_2_-induced AKT and β-catenin phosphorylation in MKN45 and AGS cells. **K**, **L**, **O**, **P** Inhibitory effect of shNCX1 on NH_4_Cl-induced AKT and β-catenin phosphorylation in MKN45 and AGS cells. (^*^P < 0.05, ^**^P < 0.01, ^***^P < 0.001, ^****^P < 0.0001, *n* = 3; ns, no significant differences).

We next elucidated NCX1-promoted oncogenic mechanisms. Since AKT/β-catenin pathway plays a crucial role in the development of GC [31] and colorectal cancer [32], and aberrant [Ca^2+^]_cyt_ promoted GC through this pathway [11], we therefore examined the role of AKT/β-catenin. First, after NCX1 was activated by CaCl_2_, both AKT phosphorylation (Ser473) and β-catenin phosphorylation (Ser675) were increased in MKN45 and AGS cells (Fig. 7A, B, E, F, I, J, M, N). Second, the CaCl_2_-induced phosphorylation of AKT and β-catenin were attenuated by either KB-R7943 (Fig. 7A, B, E, F) or NCX1 knockdown (Fig. 7I, J, M, N). Third, NH_4_Cl also increased AKT phosphorylation (Ser473) and β-catenin phosphorylation (Ser675) in MKN45 and AGS cells (Fig. 7C, D, G, H, K, L, O, P), which were attenuated by KB-R7943 (Fig. 7C, D, G, H) and NCX1 knockdown (Fig. 7K, L, O, P). Therefore, both CaCl_2_- and NH_4_Cl-induced NCX1 activation could stimulate phosphorylation of AKT and β-catenin in GC cells.

### NCX1 couples with TRPC1 to promote GC through AKT pathway

**Fig. 8:**
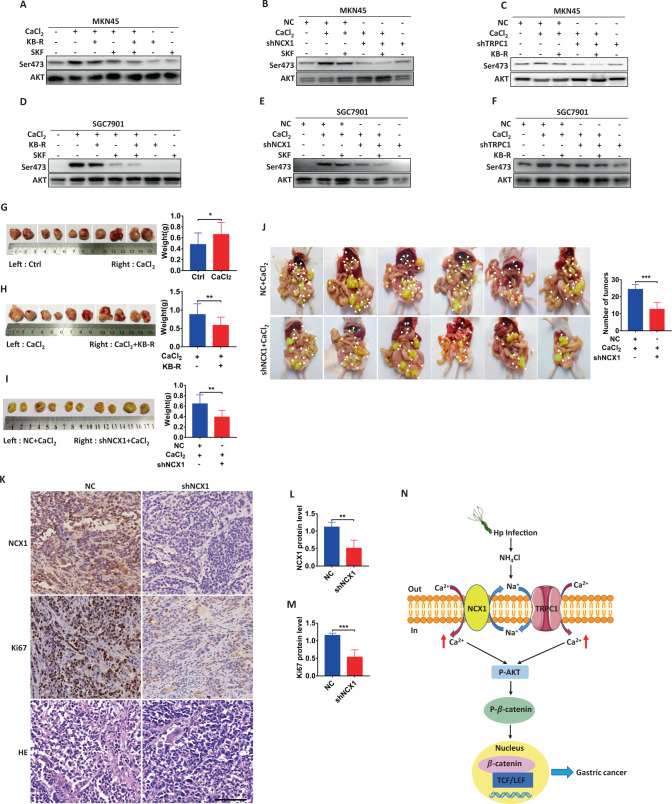
TRPC1/NCX1 coupling induces AKT phosphorylation and promotes GC growth and metastasis. **A**, **D** Inhibitory effects of either KB-R7943 (KB-R, 1 μM in MKN45, 8 μM in SGC7901), SKF96365 (SKF, 1 μM) or KB-R plus SKF on CaCl_2_ (2 mM)-induced AKT phosphorylation in GC cells. **B**, **E** Inhibitory effects of SKF, shNCX1 or shNCX1 plus SKF on CaCl_2_ (2 mM)-induced AKT phosphorylation in GC cells. **C**, **F** Inhibitory effects of either KB-R, shTRPC1 or shTRPC1 plus KB-R on CaCl_2_-induced AKT phosphorylation in GC cells. CaCl_2_ promoted growth of xenografted gastric tumors (**G**), which was attenuated by either KB-R7943 (**H**) or shNCX1 (**I**). **J** Inhibitory effects of shNCX1 on CaCl_2_-induced gastric tumor metastasis. **K** Immunohistochemical analysis and histological examination on expression of NCX1 and Ki67 proteins with or without NCX1 knockdown in GC tissues. Scale bar = 100 μm for each image. **L**, **M** Summary data comparing expression of NCX1 and Ki67 proteins analyzed by immunohistochemistry between with or without NCX1 knockdown in GC tissues. (^*^P < 0.05, ^**^P < 0.01, ^***^P < 0.001, ^****^P < 0.0001, *n* = 3; ns, no significant differences). **N** The proposed oncogenic mechanisms of TRPC1/NCX1 coupling via Ca^2+^/AKT/β-catenin pathway in *Hp*-associated GC.

Since the Ca^2+^ entry mode of NCX1 usually functions via a coupling with TRPC1 [29], we investigated whether TRPC1 channels are involved in NCX1-mediated AKT phosphorylation. Western blotting analysis exhibited that after CaCl_2_ induced AKT phosphorylation in GC cells, either NCX1 inhibitor KB-R7943 or TRPC1 inhibitor SKF96365 significantly attenuated the CaCl_2_-induced AKT phosphorylation; but both of them further attenuated it (Fig. 8A, D). Moreover, either a combination of shNCX1 and SKF96365 (Fig. 8B, E) or a combination of shTRPC1 and KB-R7943 (Fig. 8C, F) further attenuated the CaCl_2_-induced AKT phosphorylation. These data verify TRPC1/NCX1 coupling enhances AKT phosphorylation in GC cells.

### NCX1 activation enhances GC growth and metastasis in vivo

We applied subcutaneously xenografted GC model of nude mice to verify the oncogenic role of the Ca^2+^ entry mode of NCX1 in GC growth in vivo. NCX1 activation by CaCl_2_ increased tumor weights (Fig. 8G), which was attenuated by KB-R7943 (Fig. 8H). Moreover, the knockdown of NCX1 in SGC-7901 cells by NCX1-shRNA lentiviruses markedly suppressed GC growth (Fig. 8I). Immunohistochemical analysis showed that the tumors derived from the implants pre-treated with NCX1-shRNA lentiviruses had lower expression of NCX1 and Ki67 than those pre-treated with control shRNA (Fig. 8K, L, M). Therefore, NCX1 promotes GC growth in vivo.

We further applied abdominal transplantation tumor model of nude mice to verify NCX1-promoted GC metastasis in vivo. As shown in Fig. 8J, CaCl_2_-induced GC metastasis was markedly suppressed by pretreatment with NCX1-shRNA lentiviruses. Compared to NC group, tumor numbers in the group pretreatment with NCX1-shRNA were decreased by about 50%. Therefore, NCX1 promotes GC metastasis in vivo as well.

## Discussion

In the present study, we demonstrate for the first time that NCX1 and TRPC1 simultaneously participate in GC development. Several lines of evidence suggest that NCX1 promotes human GC growth and metastasis by a novel coupling to TRPC1 channels. First, the expression of NCX1 and TRPC1 was enhanced in human primary GC tissues and most GC cell lines. Second, the enhanced NCX1 expression was closely correlated with poor progression and survival of GC patients. Third, NCX1 and TRPC1 were co-expressed in parallel, co-localized and bound on the membrane of GC cells. Forth, co-stimulation of NCX1 and TRPC1 with CaCl_2_ and *Hp* virulence factors promoted GC cell proliferation, migration and invasion in vitro, and increased gastric tumor size, number and peritoneal dissemination in vivo. Fifth, by coupling with TRPC1, NCX1 operated in Ca^2+^ entry mode to promote GC through AKT/β-catenin signaling pathway.

NCX1 plays a critical role in mediating [Ca^2+^]_cyt_ homeostasis in various types of human cells [12]; however, it was investigated predominately in the cardiovascular, nervous, and renal systems instead of GI tract [13]. Nothing is currently known about NCX1 in gastric epithelia even though it is expressed in gastric smooth muscle to likely mediate motility [33]. We revealed previously that NCX1 not only physiologically mediates lower esophageal sphincter relaxation [34] and intestinal epithelial ion transports [35], but also pathologically involves in GI inflammation and cancer [18, 36]. Disruption of [Ca^2+^]_cyt_ homeostasis induced by the enhanced Ca^2+^ entry mode of NCX1 has been detected in several cancer, such as pancreatic cancer [36], breast cancer [37], glioblastoma [38], and melanoma [39]. Although we have demonstrated the roles of NCX1-mediated Ca^2+^ signaling in esophageal cancer [18], pancreatic cancer [36] and hepatocellular carcinoma [19], the pathological roles of NCX1 in the stomach, especially in GC development remain totally unexplored. In the present study, we verified the enhanced NCX1 expression in human primary GC tissues and cells; and the enhanced NCX1 expression was correlated with larger tumor size, higher histological grade, lymphatic metastasis, advanced clinical stage and poor prognosis, which strongly suggests NCX1 as a potential marker for GC prognosis.

We have provided experimental data to support a pivotal oncogenic role of NCX1 in *Hp*-associated GC since its activation with either calcium or *Hp* virulence factors promoted GC cell proliferation, invasion and metastasis in vitro and in vivo. Consistently, both selective blocker for the Ca^2+^ entry mode of NCX1 and its specific knockdown attenuated the oncogenic effects of NCX1. Therefore, NCX1 may play a general oncogenic role in GI cancer, such as in GC reported here and in esophageal cancer [18], pancreatic cancer [36] and hepatocellular carcinoma [19] reported previously. Moreover, TRP channels also play different roles in GC development [9–11]. Although the enhanced TRPC1 is likely involved in GC progression [24], its association with NCX1 is unknown. Here we revealed not only enhanced TRPC1 expression but also its co-localization and binding with NCX1 in human GC cells. Importantly, TRPC1-promoted GC cell proliferation and migration were attenuated by TRPC1 channel blocker. Therefore, in parallel with NCX1, TRPC1 also plays an oncogenic role in GC.

Under physiological status, NCX1 primarily functions in Ca^2+^ exit mode; however, under some pathological conditions (such as in tumorigenesis), NCX1 is switched to Ca^2+^ entry mode to allow sustained Ca^2+^ entry [12]. Most reports have suggested TRPC as a potential partner for NCX mode switch in non-excitable cells (such as GC cells) [29]. Na^+^ could enter through TRPC channels to raise [Na^+^]_cyt_ under the restricted membrane space and induce membrane depolarization, switching NCX1 to Ca^2+^ entry mode [29]. Indeed, in the present study we revealed a novel coupling of TRPC1 and the Ca^2+^ entry mode of NCX1 in GC development because: 1) both NCX1 and TRPC1 play similar oncogenic roles in GC; 2) GC cell proliferation and migration could be further attenuated by a combination of selective blockers and specific knockdown of NCX1 and TRPC1; 3) CaCl_2_ and *Hp* virulence factors could stimulate TRPC1 and NCX1 coupling to induce Ca^2+^ signaling; 4) a protein-protein interaction of TRPC1 and NCX1 is verified in GC cells. Therefore, due to a general existence of TRPC and NCX1 coupling in GC, pancreatic cancer [23], and hepatocellular carcinoma [19], this coupling could allow aberrant sustained Ca^2+^ entry to promote most digestive cancer.

It has been well documented that aberrant Ca^2+^ signaling participates in chronic inflammation and cancer, such as GC developed from *Hp*-associated gastric inflammation [40]. Moreover, Iimuro et al. found that dietary calcium enhances the *Hp*-induced gastritis in Mongolian gerbils [41]; in contrast, calcium channel blockers attenuate chemically induced gastritis and GC in rats [42, 43]. Consistently with our previous report on the oncogenic role of calcium in GC development [11], here we further reveal that CaCl_2_ and *Hp* virulence factors enhance the expression, activity and coupling of NCX1 and TRPC1 to promote GC development, strongly suggesting a critical role of TRPC1/NCX1-mediated aberrant Ca^2+^ signaling in *Hp*-associated GC.

Our results indicate the TRPC1/NCX1-mediated Ca^2+^ signaling increases AKT and β-catenin phosphorylation in GC cells, which supports our notion that TRPC1/NCX1 coupling induces GC development through the Ca^2+^/AKT/β-catenin pathway (Fig. 8N), further confirming the pivotal role of this pathway in GC as in our previous report [11]. Therefore, our findings strongly suggest not only that aberrant Ca^2+^ entry could promote GC via Ca^2+^/AKT/β-catenin pathway, but also that calcium supplement and *Hp* infection are likely synergistic risk factors for GC pathogenesis.

In conclusion, we demonstrate for the first time that TRPC1/NCX1 coupling promotes *Hp*-associated GC development. Mechanistically, TRPC1/NCX1 coupling-mediated aberrant Ca^2+^ entry activates AKT/β-catenin pathway to consequently promote GC. Although NCX and TRP channels represent a relatively new field of cancer research with most studies still in their infancy, they hold tremendous potentials that have yet to be uncovered in the hopes of achieving major clinical breakthroughs in GC therapy. Particularly, due to a critical role of the Ca^2+^ exit mode of NCX1 under physiological status, targeting TRPC1/NCX1 coupling could be a novel strategy for selectively blocking the Ca^2+^ entry mode of NCX1 to potentially treat if not all solid cancers but at least digestive cancer with less side effect.

## Materials and methods

### Ethics statement and human tissue samples

All animal and clinical studies were approved by the Clinical Research Ethics Committee of the Qingdao University Medical College, Qingdao and Army Medical University (AMU), Chongqing, China. 52 pairs of GC and adjacent tissues for western blotting were collected from the surgical patients in Xinqiao Hospital of the AMU and all resected specimens were confirmed by pathological examination. Informed consent was obtained for all patients. All animal care and experimental procedures complied with the “Guide for the Care and Use of Laboratory Animals” published by the National Institutes of Health, USA. Animal studies are reported in compliance with the ARRIVE guidelines [44].

### Cell culture

The human gastric normal epithelial mucosa cell line (GES1) and gastric cancer cell lines MKN45, SGC7901, AGS, BGC803, BGC823 and SNU216 were purchased from Chinese Academy of Sciences (Shanghai, China). The CHO cells with NCX1 overexpression and CHO-K1 cells without NCX1 overexpression were kindly provided by the University of California, San Diego, California, USA [34]. All cells were maintained in RPMI-1640, DMEM-HIGH GLUCOSE or F-12 medium (HyClone, USA) supplemented with 10% fetal bovine serum (HyClone, USA) and 1% penicillin/streptomycin (Invitrogen, USA) in a 37 °C humidified atmosphere containing 5% CO_2_.

### Preparation and infection of lentiviruses

Lentiviruses were purchased from HANBIO (Shanghai, China). The sequences for NCX1 shRNA, TRPC1 shRNA and NC were described in the supplemental materials. GC cells were infected with lentiviruses according to the protocol of the manufacturer.

### Quantitative real-time PCR

Quantitative real-time PCR was performed as previously described [9, 10]. All samples were run in triplicate, and β-actin was used as an internal control. Primers were described in the supplemental materials.

### Western blotting

Western blotting was performed as previously described [9, 10]. The antibodies were described in the supplemental materials.

### Co-immunoprecipitation and immunohistochemistry

Co-immunoprecipitation [45, 46] and immunohistochemistry [9, 11] were performed as previously described. The antibodies of co-immunoprecipitation are anti-TRPC1 (No. ACC-010, alomone labs, Israel) and anti-NCX1 (No. ANX-011, alomone labs, Israel). The GC and adjacent tissue microarray for immunostaining were purchased from SHANGHAI OUTDO BIOTECH CO., LTD (Shanghai, China). The tissue samples were incubated with anti-NCX1 (No. N216, Sigma, USA). The degree of staining in the NCX1 sections was observed and scored by two pathologists. According to previously defined criteria by T. Takenoue et al. [47], the percentage of NCX1 positivity was scored from 0 to 4 as follows: 0, <5%; 1, 5–25%; 2, 26–50%; 3, 51–75%; 4, >=76%. The intensity of immunostaining was scored as: 0 (no staining); 1 (weak); 2 (moderate); and 3 (intense). Subsequently, the two scores were multiplied and the product was defined as immunohistochemical score. Accordingly, the final expression level of NCX1 was defined as low (0–4) and high (5–12). The IHC of mice tissue samples were incubated with anti-NCX1 (No. ANX-011, alomone labs, USA) and anti-Ki67 (No. ab15580, Abcam, UK), and the staining section were performed as previously described [10].

### Immunofluorescence assay

After fixed and blocked, the GC cells were incubated with anti-NCX1 antibody (No. MA3-926, Invitrogen) overnight at 4 °C. Next, the cells were incubated with Cy3 labeled anti-mouse (No. A0521, Beyotime, China) secondary antibody for 1 h at room temperature. Then cells were incubated with anti-TRPC1 antibody (No. ACC-010, alomone labs, Israel) and 647 labeled anti-rabbit (No. A0468, Beyotime, China) secondary antibody for 1 h at room temperature respectively. Finally, nuclei were stained with DAPI for 5 min and images were captured using confocal microscope.

### Cell proliferation and scratch assays

Cell proliferation assay was performed as previously described [9]. Cell proliferation was measured by CCK8 assay (No. C0038, Beyotime Biotechnology, China) according to the protocol of the manufacturer. Cell scratch assay was performed as previously described [48]. After scratching, gently wash the cell monolayer to remove detached cells. Then, replenish with serum free medium containing different drugs. 0 and 24 h take photos respectively.

### Transwell migration and invasion assays

Transwell migration and invasion assays were performed as previously described [9]. Cells were cultured upper chamber with 200 μL serum-free medium containing with different drugs. The lower chambers were filled with 600 μL medium plus 10% FBS. For invasion assays, the upper surface of the polycarbonate filter was coated with 10% Matrigel (Collaborative Biomedical, USA). The cells were treated for 24 h.

### Measurement of [Ca^2+^]_cyt_ by digital Ca^2+^ imaging

[Ca^2+^]_cyt_ imaging experiments were performed as previously described [49, 50]. The [Ca^2+^]_cyt_ imaging solution were described in the supplemental materials.

### Tumor xenograft and peritoneal dissemination assays in nude mice

Tumor xenograft assay was performed as previously described [11]. The male nude mice were purchased from Vital River Laboratory Animal Technology Co., Ltd (Beijing, China). Randomization and single-blinding were used for the measurement. After tumor sizes grow to 1 mm^3^, CaCl_2_ (4 mM), KB-R7943 (30 μM), or CaCl_2_ plus KB-R7943 were injected into the tumors in one side of the armpits once a day, and 0.1% DMSO into the other side as controls. Similarly, the shNCX1 SGC7901 cells or negative control NC was separately injected into each side, and CaCl_2_ was injected into the tumors of each side once a day. Four weeks later, xenografted tumors were quantified. For the peritoneal dissemination assay, 1 × 10^6^ shNCX1 SGC7901 cells and the NC cells were injected into the abdominal cavity of nude mice. CaCl_2_ (4 mM) was injected into abdominal cavity once a day. Five weeks later, xenografted tumors were quantified.

### Statistical analysis

SPSS Statistics 26.0 (RRID:SCR_002865, USA) and GraphPad Prism 7.0 (RRID:SCR_002798, USA) software were used to analyze the data. All data shown are means ± SD. All experiments were repeated for at least three times. Student’s unpaired, two-tailed t test or one-way ANOVA were used to analyze statistical significance differences of experimental groups. The patient survival was examined by the log-rank test using the Kaplan-Meier method. Significant differences (^*^P < 0.05) are expressed in the figures and figure legends.

## Supplementary information

Supplementary materials

Supplementary figure 1-5

Supplementary table 1
