# Supplementary material for: NCX1 coupled with TRPC1 to promote gastric cancer via Ca2+/AKT/β-catenin pathway
Source: Oncogene. 2022 Jul 26;41(35):4169–82. doi: 10.1038/s41388-022-02412-9 (PMC9418000; doi:10.1038/s41388-022-02412-9)
Supplement: Supplementary file 1 — Supplementary materials [file 41388_2022_2412_MOESM1_ESM.docx]

1. **Supplementary figure legends and table title**

**Supplementary Figures**

**Supplementary figure 1. The effect of SN-6 and SAR7334 on CaCl_2_ (1 mM)-induced** **proliferation of GC cells. (A-B)** Inhibitory effect of SN-6 (10 μM) on CaCl_2_-induced proliferation of MKN45 and AGS cells. **(C-D)** SAR7334 (100 nM) did not affect CaCl_2_-induced proliferation of MKN45 and AGS cells.

**Supplementary figure 2. Dose-dependent inhibition of KB-R7943 and SKF96365 on proliferation of human GC cells and CHO-NCX1 cells**

**(A-D)** Dose-dependent inhibition of KB-R7943 on proliferation of MKN45 (A), AGS (B), SGC7901 (C) and CHO-NCX1 cells (D). **(E-G)** Dose-dependent inhibition of SKF96365 on proliferation of MKN45 (E), AGS (F), and SGC7901 cells (G). (**P* < 0.05, ***P* < 0.01, ****P* < 0.001, *****P* < 0.0001, n = 3; ns, no significant differences).

**Supplementary figure 3. NCX1 activation promotes migration of human GC cells**

**(A-B)** Inhibitory effect of KB-R7943 (KB-R, 1 μM in MKN45, 4 μM in AGS) on CaCl_2_-induced migration of MKN45 and AGS cells analyzed by cell scratch assay. Scale bar = 200 μm for each image. **(C-D)** Inhibitory effect of KB-R7943 on CaCl_2_-induced migration of MKN45 and AGS cells analyzed by transwell migration assays. Scale bar = 200 μm for each image. (**P* < 0.05, ***P* < 0.01, ****P* < 0.001, *****P* < 0.0001, n = 3; ns, no significant differences).

**Supplementary figure 4. CaCl_2_ and NH_4_Cl promote migration of human GC cells through NCX1 activation**

**(A-C)** Inhibitory effect of shNCX1 on CaCl_2_-induced migration of MKN45, AGS and SGC7901 cells analyzed by transwell migration assays. **(D-F)** Inhibitory effect of shNCX1 on NH_4_Cl-induced migration of MKN45, AGS and SGC7901 cells. Scale bar = 200 μm for each image. (**P* < 0.05, ***P* < 0.01, ****P* < 0.001, *****P* < 0.0001, n = 3; ns, no significant differences).

**Supplementary figure 5. No effects of CaCl_2_, NH_4_Cl, LPS and *H. pylori* on NCX1 protein expression in GES1 cells**

**(A-D)** Representative and summary data showing no effects of CaCl_2_ (2 mM)-, NH_4_Cl (2 mM)-, LPS (10 ng/ml)- and *H. pylori*-induced protein expression of NCX1 in GES1 cells in different time courses. (n = 3; ns, no significant differences).

**Supplementary table 1. Correlation between the NCX1 expression and clinicopathological characteristics in the patients (n=<80) with GC.**

1. **The sequences of shRNA**

The sequences for NCX1 shRNA, TRPC1 shRNA and NC were as follows: shNCX1-1 (5′- GCTAGGATTCTGAAGGAACTT-3′), shNCX1-2 (5′- CATCTGGAGCTCGAGGAAATGTTAT-3′), shTRPC1 (5′- GCTAAGGATTTACTTGCACAA-3′), and NC shGFP (5′- TTCTCCGAACGTGTCACGTAA-3′).

1. **The primers**

Primers for TRPC1 and β-actin were as follows: TRPC1: 5′-AGGATAGCCTCCGGCATTC-3′, TRPC1: 3′-TTCCACCTCCACAAGACTTAGT-5′; β-actin: 5′-GGCATCCACGAAACTACCTT-3′, β-actin: 3′-TCGTCCTCATACTGCTCAGGC -5′.

1. **Western blotting antibodies**

The following antibodies were used for Western blotting: anti-NCX1, 1:1,000 (No. ab177952, Abcam, UK), anti-TRPC1, 1:200 (No. ACC-010, alomone labs, Israel), anti-β-catenin, 1:1000 (No. 8480, Cell Signaling Technology, USA), anti-phospho-β-catenin, 1:1000 (No. 9567, Cell Signaling Technology, USA), anti-AKT, 1:1000 (No. 4691, Cell Signaling Technology, USA), anti-phospho-AKT, 1:1000 (No. 4051, Cell Signaling Technology, USA), and anti-GAPDH, 1:10,000 (No. 60004-1-Ig, Proteintech, USA).

1. **The solutions for [Ca^2+^]_cyt_ imaging**

The physiological salt solutions (PSS) contained the following: 140 mM Na^+^, 5 mM K^+^, 2 mM Ca^2+^, 147 mM Cl^-^, 10 mM HEPES and 10 mM glucose (pH 7.4). The 0 Na^+^ solution (0 Na^+^) contained the following: 140 mM Li^+^, 5 mM K^+^, 2 mM Ca^2+^, 147 mM Cl^-^, 10 mM HEPES and 10 mM glucose (pH 7.4). The 0 Ca^2+^ solution (0 Ca^2+^) contained the following: 140 mM Na^+^, 5 mM K^+^, 145 mM Cl^-^, 0.5 mM EGTA, 10 mM HEPES and 10 mM glucose (pH 7.4). The 0 Na^+^ and 0 Ca^2+^ solution (0 Na^+^-0 Ca^2+^) contained the following: 140 mM Li^+^, 5 mM K^+^, 145 mM Cl^-^, 0.5 mM EGTA, 10 mM HEPES and 10 mM glucose (pH 7.4).
