# Supplementary material for: NCX1 coupled with TRPC1 to promote gastric cancer via Ca2+/AKT/β-catenin pathway
Source: Oncogene. 2022 Jul 26;41(35):4169–82. doi: 10.1038/s41388-022-02412-9 (PMC9418000; doi:10.1038/s41388-022-02412-9)

Supplementary figure 1.

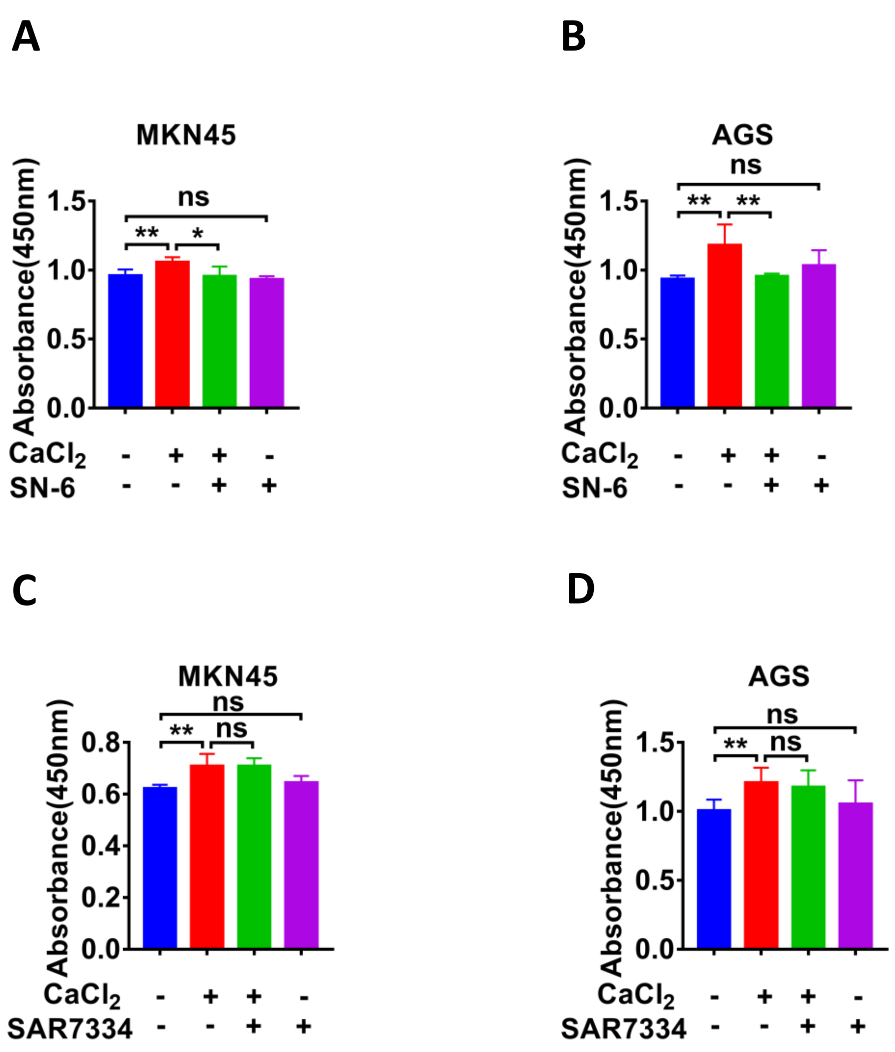

Supplementary figure 2.

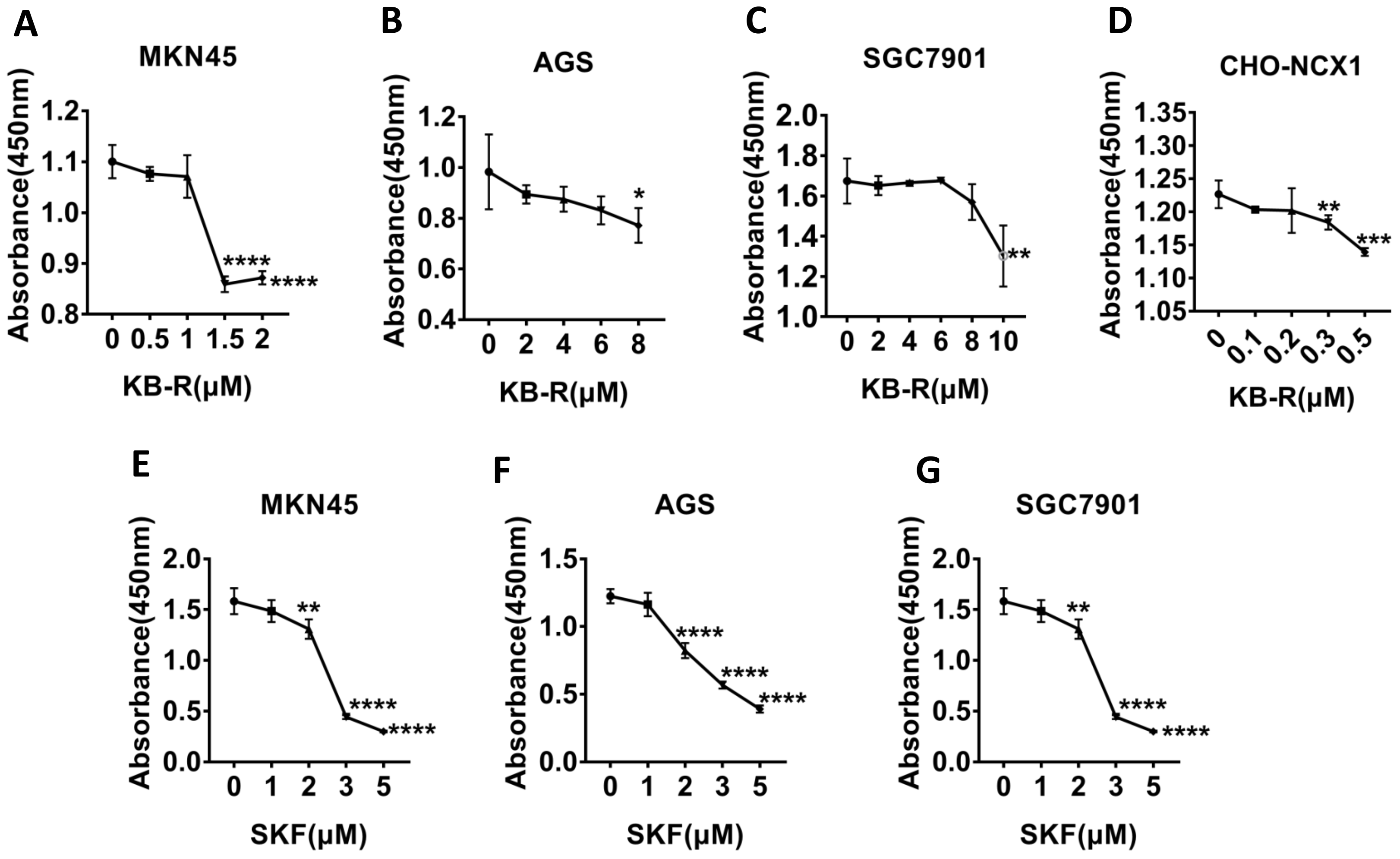

Supplementary figure 3.

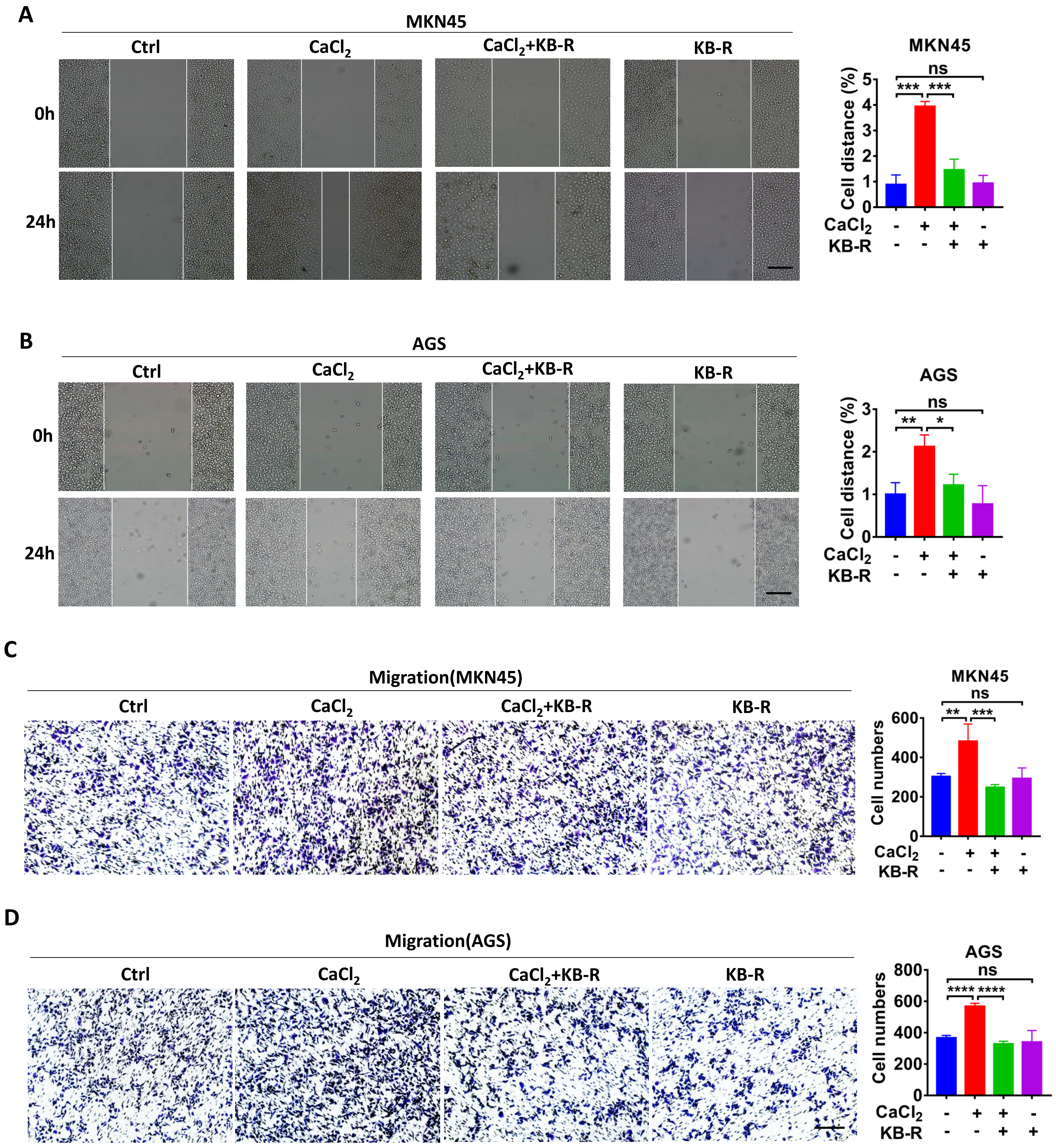

Supplementary figure 4.

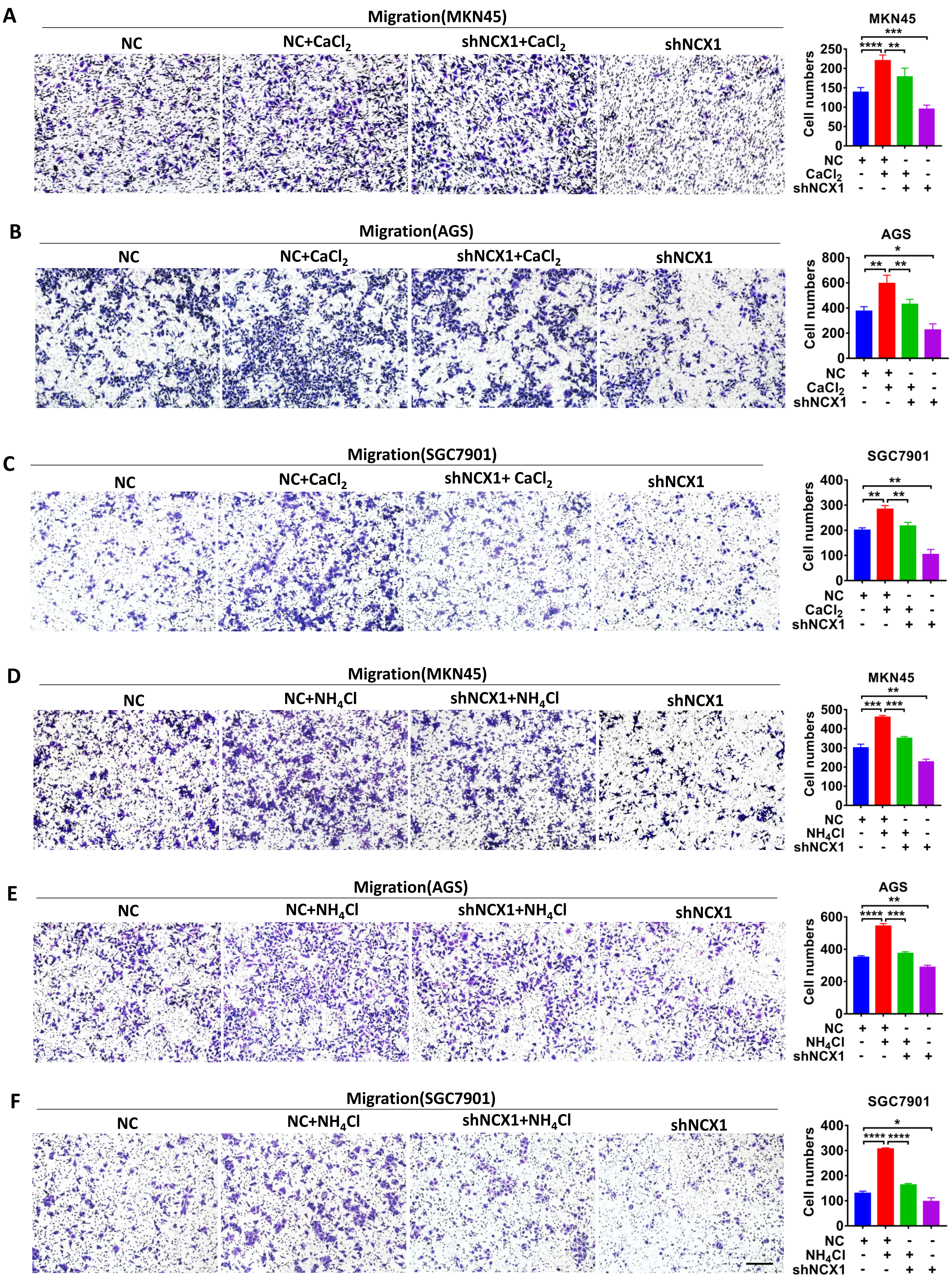

Supplementary figure 5.

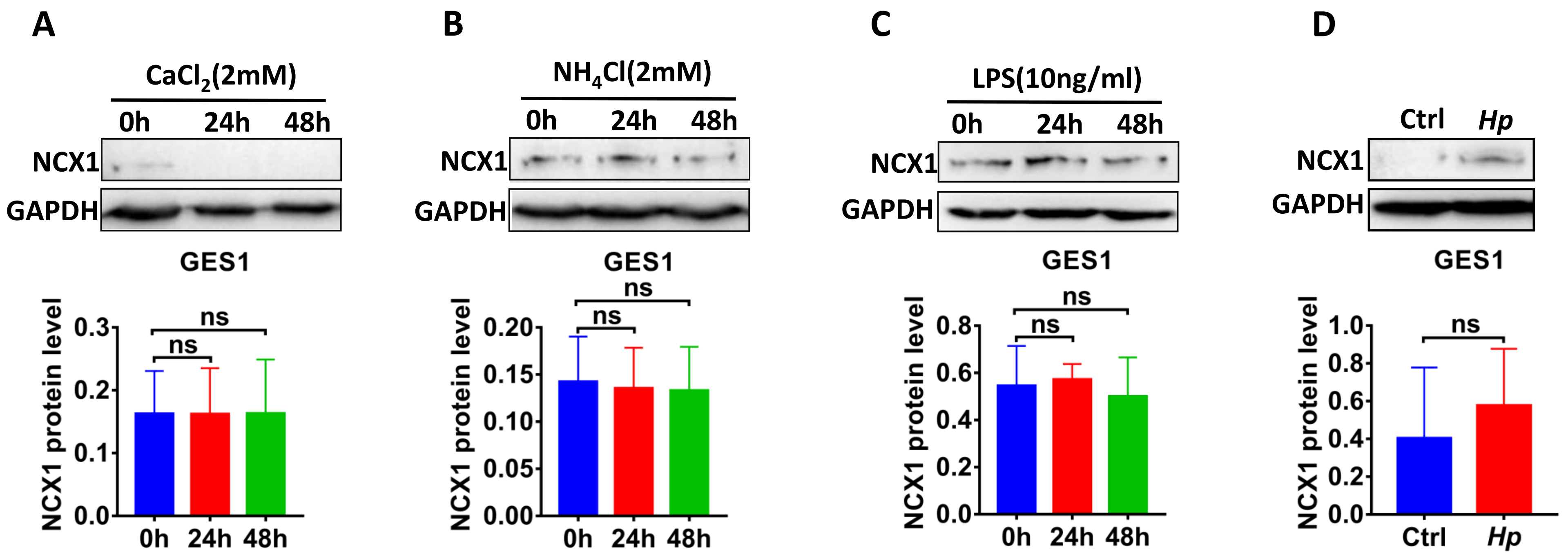

Supplement: Supplementary file 2 — Supplementary figure 1-5 [file 41388_2022_2412_MOESM2_ESM.pdf]
