# Supplementary material for: NCX1 coupled with TRPC1 to promote gastric cancer via Ca2+/AKT/β-catenin pathway
Source: Oncogene. 2022 Jul 26;41(35):4169–82. doi: 10.1038/s41388-022-02412-9 (PMC9418000; doi:10.1038/s41388-022-02412-9)
Supplement: Supplementary file 3 — Supplementary table 1 [file 41388_2022_2412_MOESM3_ESM.pdf]

Supplementary table 1. Correlation between the NCX1 expression and clinicopathological characteristics in the patients (n=<80) with GC.

Characteristic	No. Patients (n=<80)	NCX1 Expression		***Chi**-square*	***P-**value*
		Low No. (%)	High No. (%)		
Age					
<60 years	21	10(47.6%)	11(52.4%)	0.173	0.677
≥60 years	59	25(42.4%)	34(57.6%)		
Gender					
Male	61	23(37.7%)	38(62.3%)	3.814	0.051
Female	19	12(63.2%)	7(36.8%)		
Tumor size					
<5 cm	31	18(58.1%)	13(41.9%)	4.214	**0.040***
≥5 cm	49	17(34.7%)	32(65.3%)		
Histological grade					
G1/2	22	12(54.5%)	10(45.5%)	0.417	0.519
G3/4	56	26(46.4%)	30(53.6%)		
Lymphatic metastasis					
Negative	21	14(66.7%)	7(33.3%)	6.077	**0.014***
Positive	59	21(35.6%)	38(64.4%)		
Clinical stage					
I/II	30	18(60%)	12(40%)	7.185	**0.007***
III/IV	45	13(28.9%)	32(71.1%)		
